# Supplementary material for: The Multifunctional Catalytic Hemoglobin from Amphitrite ornata: Protocols on Isolation, Taxonomic Identification, Protein Extraction, Purification, and Characterization
Source: Methods Protoc. 2024 Dec 11;7(6):100. doi: 10.3390/mps7060100 (PMC11678344; doi:10.3390/mps7060100)
Supplement: Supplementary file 1 [file mps-07-00100-s001.zip › mps-3355804-supplementary.pdf]

Supplementary Information

The Multifunctional Catalytic Hemoglobin from *Amphitrite ornata*: Protocols on Isolation, Taxonomic Identification, Protein Extraction, Purification, and Characterization

Anna L. Husted [1], Victoria R. Sutton [1], Lauren A. Presnar [1], R. Kevin Blackburn [2], Joseph L. Staton [1], Stephen A. Borgianini [1] and Edward L. D'Antonio [1,*]

[1] Department of Natural Sciences, University of South Carolina Beaufort, 1 University Boulevard, Bluffton, SC 29909, USA;
[2] Department of Molecular and Structural Biochemistry, North Carolina State University, 120 W Broughton Drive, Raleigh, NC 27607, USA
* To whom correspondence should be addressed:

Prof. Edward L. D'Antonio, Ph.D.
Department of Natural Sciences
University of South Carolina Beaufort
1 University Boulevard
Bluffton, South Carolina 29909
United States of America
Tel.: +1 843-208-8101
E-mail: edantonio@uscb.edu

Table of Contents

I. Relevant GenBank Entries

a. Nucleotide sequence for *Amphitrite ornata* mitochondrial cytochrome c oxidase subunit I (*Ao*COI) (GenBank accession no. OQ322956.1)….…………**S3**

b. Amino acid sequence for *Amphitrite ornata* dehaloperoxidase isoenzyme A (*Ao*DHP-A) (GenBank accession no. AAF97245.1)………..……**S4**

I. Relevant GenBank Entries

a. Nucleotide sequence for *Amphitrite ornata* mitochondrial cytochrome c oxidase subunit I (*Ao*COI) (GenBank accession no. OQ322956.1)

Amphitrite ornata isolate SERCINVERT2482 cytochrome oxidase subunit 1 (COI) gene, partial cds; mitochondrial

GenBank: OQ322956.1

Number of Nucleotide Bases: 658

Source: https://www.ncbi.nlm.nih.gov/nuccore/OQ322956.1

FASTA Format

```
>OQ322956.1 Amphitrite ornata isolate SERCINVERT2482 cytochrome oxidase subunit 1 (COI) gene, partial cds; mitochondrial
AACTCTATATTTTATTTTTGGTATTTGAGGAGGCTTATTAGGAACCTCCATAAGATTACTAATTCGAA
TTGAACTTGGACAACCAGGAGCTTTTCTTGGAAGAGATCAACTGTATAACACAGTAGTAACAGCCCAT
GGTCTACTTATAATTTTTTTTCTAGTTATACCGATCCTTATTGGGGGATTCGGAAATTGATTACTACC
TCTTATATTAGGAGCACCCGATATAGCTTTCCCACGAATAAATAATATAAGATTTTGATTTTTACCTC
CTGCCCTTCTTCTATTACTTAGTTCAGCAGCTGTAGAAAAAGGTGTAGGTACAGGATGAACTGTGTAT
CCTCCTTTATCAAGAAATCTAGCACACGCTGGCCCCTCTGTAGATCTAGCTATTTTTTCCCTACATTT
AGCTGGGATCTCCTCAATCCTAGGAGCAATTAATTTTATTACAACAGTAGCTAACATACGATGAAAAG
GACTACGACTAGAACGAATCCCTCTATTTGTTTGAGCAGTTAATATTACTGTTATTTTACTTCTATTA
TCCTTACCAGTTCTAGCTGGTGCAATCACTATATTATTAACAGACCGTAATGTTAATACTTCTTTCTT
TGACCCATCAGGGGGAGGGGACCCAATTCTTTATCAACACTTATTT
```

b. Amino acid sequence for *Amphitrite ornata* dehaloperoxidase isoenzyme A (*Ao*DHP-A) (GenBank accession no. AAF97245.1)

Amphitrite ornata dehaloperoxidase A

GenBank: AAF97245.1

Number of Amino Acids: 138

Source: https://www.ncbi.nlm.nih.gov/protein/AAF97245.1

FASTA Format

>AAF97245.1 dehaloperoxidase A [*Amphitrite ornata*]

MGFKQDIATIRGDLRTYAQDIFLAFLNKYPDERRYFKNYVGKSDQELKSMAKFGDHTEKVFNLMMEVA

DRATDCVPLASDANTLVQMKQHSSLTTGNFEKLFVALVEYMRASGQSFDSQSWDRFGKNLVSALSSAG

MK
